# Der Einsatz von Sprach- und Videotelefonie in der Psychotherapie als Chance und Herausforderung: Eine Querschnitterhebung zu zwei Erhebungszeitpunkten

**DOI:** 10.1007/s00729-021-00187-0

**Published:** 2021-12-14

**Authors:** Christian Korunka, Claudia Höfner, Magdalena Straßer, Markus Hochgerner, Gerd Mantl

**Affiliations:** 1 Fakultät für Psychologie, Institut für Arbeits‑, Wirtschafts- und Sozialpsychologie, Universität Wien, Universitätsstraße 7, 1010 Wien, Österreich; 2 Österreichischer Arbeitskreis für Gruppentherapie und Gruppendynamik, Lenaugasse 3, 1080 Wien, Österreich

**Keywords:** Video-Psychotherapie, Internetgestützte Psychotherapie, Covid-19, Digitalisierung, Therapeutische Beziehung, Video psychotherapy, Covid-19, Digitalization, Therapeutic relationship

## Abstract

Die Video-Psychotherapie, als eine Form der internetgestützten Psychotherapie, hat sich in den letzten Jahrzehnten langsam verbreitet. Die im letzten Jahrzehnt veröffentlichten Reviews und erste Meta-Analysen zeigen die Möglichkeiten dieser digitalisierten Form der Psychotherapie auf. Die Covid-19 Pandemie und die Anforderungen der Lockdowns haben diese Entwicklung rasch beschleunigt.

Diese Studie systematisiert die Erfahrungen österreichischer Psychotherapeut_innen mit Video-Psychotherapie im ersten Jahr der Pandemie, indem sie einen Querschnitt zu zwei Erhebungszeitpunkten abbildet. Im ersten Lockdown nahmen 743, im zweiten Lockdown 212 Psychotherapeut_innen an der Studie teil. Inhaltsanalytische Auswertungen zeichnen ein differenziertes Bild der Möglichkeiten und Grenzen von Video-Psychotherapie, und auch eine intensive Lernerfahrung im ersten Jahr der Pandemie. Die Ergebnisse werden vor dem Hintergrund der vier therapeutischen Strömungen diskutiert, Grenzen und Möglichkeiten dieser Form der Psychotherapie differenziert aufgezeigt.

## Abstract (en)

Video psychotherapy, as a form of Internet-based psychotherapy, has slowly spread over the last decades. Reviews and initial meta-analyses published in the last decade demonstrate the potential of this digitized form of psychotherapy. The Covid-19 pandemic and lockdown requirements have rapidly accelerated this development.

This study systematizes Austrian psychotherapists’ experiences with video psychotherapy in the first year of the pandemic by mapping a cross-section at two measurement time points. In the first lockdown, 743 psychotherapists participated in the study, in the second lockdown 212. Content analytic evaluations paint a nuanced picture of the possibilities and limitations of video psychotherapy, and also an intensive learning experience in the first year of the pandemic. The results are discussed against the background of the four therapeutic streams, and the limits and possibilities of this form of psychotherapy are differentiated.

## Einleitung

Psychotherapie ist, so wie viele andere Arbeitsbereiche, von der umfassenden Digitalisierung unserer Lebenswelten erfasst. Aufgrund des hohen Stellenwerts „analoger“ Konzepte wie beispielsweise der personalen Begegnung in der therapeutischen Beziehung im physischen Raum, hatte die Digitalisierung im Vergleich zu anderen Berufsgruppen allerdings bisher einen geringen Stellenwert. Nur wenige Psychotherapeut_innen setzten bisher neue Kommunikationsmedien (insbesondere Video-Psychotherapie) in ihrer therapeutischen Arbeit ein. Die Covid-19 Pandemie hat dies jedoch massiv verändert. Psychotherapeut_innen waren, defacto über Nacht, gefordert ihre Arbeit in hohem Maße mit neuen Kommunikationsmedien, insbesondere mit Formen der Video-Psychotherapie (bzw. in einigen Fällen auch über Sprachtelefonie), umsetzen. Die Pandemie bot damit einen „erzwungenen“ Erfahrungsraum für die Arbeit mit Kommunikationsmedien über Distanz. Die hier vorgestellte Studie verfolgt das Ziel, in einem qualitativen Ansatz diese Erfahrungen im ersten Jahr der Pandemie zu systematisieren.

### Zur Entwicklung der Video-Psychotherapie

Video-Psychotherapie hat bereits eine rund 60-jährige Geschichte; die erste Publikation – damals wurde Video-Psychotherapie noch mit analoger TV-Röhrentechnik durchgeführt – wurde 1961 (sogar aus dem Bereich der Gruppenpsychotherapie) veröffentlicht (Wittson et al. 1961). In den darauffolgenden vier Jahrzehnten finden sich nur vereinzelte Studien zu diesem Thema. Erst mit der Jahrtausendwende und den erweiterten digitalen Möglichkeiten wurde das Thema breiter wissenschaftlich diskutiert, erste Reviews und Metaanalysen werden nun veröffentlicht (z. B. Simpson 2009). Weitere Reviews und Überblickarbeiten folgten rasch (z. B. Backhaus et al. 2012; Norwood et al. 2018; Kocsis und Yellowlees 2018). Die Befundlage auf der Grundlage dieser Arbeiten kann wie folgt zusammengefasst werden: Die Qualität der empirischen Studien ist heterogen; oft basieren sie auf kleinen Stichproben und nur in wenigen Fällen wurden die Studien nach dem RCT-Standard (randomized control trials) durchgeführt. Die Wirksamkeit von Video-Psychotherapie kann – vor diesem Hintergrund – insgesamt zumindest als vorläufig bestätigt angesehen werden, in vielen Studien durchaus mit vergleichbaren Effektstärken zu herkömmlicher Präsenz-Psychotherapie. Die Qualität der therapeutischen Beziehungen wird meist (nur) über die „Working Alliance“ erfasst, wobei einige der Studien diese als gleichwertig betrachten, in einigen anderen wird auch eine mögliche Verschlechterung diskutiert (z. B. Norwood et al. 2018). Störungsspezifische Studien mit Video-Psychotherapie wurden vorwiegend in den Bereichen Trauma, Depression und Angststörungen (z. B. Berryhill et al. 2019; Lamb et al. 2019; Rees und MacLaine 2015), meist mit guten Effektstärken, publiziert.

Eine Betrachtung nach den vier psychotherapeutischen Grundströmungen zeigt folgendes Bild: Nur wenige Studien finden sich im Bereich der humanistischen Psychotherapien. Hier bestehen, vermutlich auch auf der Grundlage von Menschenbild und dem besonderen Stellenwert der therapeutischen Beziehung, wohl die größten Vorbehalte gegenüber Video-Psychotherapie. Historisch findet sich hier allerdings eine verstärkte Auseinandersetzung mit dem Medium Telefon; So hat die Personzentrierte Psychotherapie eine starke Tradition im Bereich der Telefonseelsorge im deutschsprachigen Raum (z. B. Overmans 2017). Auch die systemische Strömung ist in den Studien nur wenig vertreten; es wurde zwar kürzlich ein systematischer Review zur Video-Psychotherapie mit Paaren und Familien veröffentlicht (de Boer et al. 2021), die überwiegende Zahl der dort vorgestellten Studien basiert allerdings auf kognitiv-behavioralen Ansätzen (Cognitive Behavioral Therapies, CBT). In den tiefenspychologischen Strömungen findet sich hingegen eine intensivere inhaltliche Auseinandersetzung, beispielsweise zu Fragen der Übertragungsbeziehung, allerdings auch nur wenige empirische Studien (z. B. Migone 2013; De Bitencoiurt Machado et al. 2016).

Die weitaus überwiegende Zahl der empirischen Studien stammt aus dem Umfeld der kognitiv-behavioralen Psychotherapien. In der bisher wohl umfangreichsten und aktuellen Metaanalyse (Fernandez et al. 2021) wird in diesem Zusammenhang euphorisch von doppelt so hohen Effektstärken von CBT-Video-Psychotherapien (g = 1,34) im Vergleich zu Nicht-CBT Video-Psychotherapien (g = 0,66) berichtet. Begründet wird dies unter anderem damit, dass die therapeutische Beziehung (die ja in der Video-Psychotherapie beeinträchtigt sein könnte) in den CBT-Ansätzen eine vergleichsweise geringe Rolle spielt. Derartige Argumentationen vernachlässigen sowohl die Erkenntnisse zum Einfluss der „therapeutic allegiance“ (vgl. Behr 2019), also die positive Verzerrung von Ergebnissen durch die Zugehörigkeit der Forscher_innen zur untersuchten Therapieströmung, als auch die zahlreichen Befunde zu den allgemeinen psychotherapeutischen Wirkfaktoren, wo insbesondere die Aspekte der therapeutischen Beziehung unumstritten den wichtigsten Faktor darstellen (vgl. Norcross und Lambert 2019). Wenn man dazu noch bedenkt, dass Video-Psychotherapie vor der Covid-19 Pandemie nur von einer relativ kleinen Gruppe an interessierten Psychotherapeut_innen angeboten wurde, dann muss wohl von einer massiven positiven Überschätzung der Effekte bei derartigen Befunden ausgegangen werden.

### Video-Psychotherapie in der Covid-19 Pandemie

Die Covid-19 Pandemie hat die Ausbildungs- und Arbeitswelt der Psychotherapie schlagartig und anhaltend verändert. Um die psychotherapeutische Versorgung aufrecht zu erhalten, mussten viele Psychotherapeut_innen gleich nach dem Beginn des ersten Lockdowns ihr Angebot über Distanz umsetzen; in vielen Fällen (insbesondere, wenn es schon zuvor Erfahrungen dazu gab) wurde dies über Telefon realisiert; die größte Umstellung betraf allerdings das – für viele oft erstmalige – Angebot von Video-Psychotherapie. Viele Psychotherapeut_innen sammelten hier erstmals Erfahrungen mit diesem Medium. Die Erfahrungen mit psychotherapeutischen Angeboten und insbesondere der Video-Psychotherapie wurden rasch in zahlreichen Publikationen und empirischen Studien systematisiert, mit meist tendenziell positiver Befundlage (Beispiele dazu: Inchausti et al. 2020; Bekes und Aafjes-van Doorn 2020; Boldrini et al. 2020). Erhebungen mit standardisierten Fragebögen erscheinen allerdings nur bedingt geeignet, die diffizilen Unterschiede, Möglichkeiten und Problemstellungen bei Video-Psychotherapie zu erfassen (siehe z. B. Probst et al. 2021). Studien mit mehreren Erhebungszeitpunkten, die auch den Erfahrungszuwachs im Bereich der Video-Psychotherapie im ersten Jahr der Covid-Pandemie thematisieren, fehlen noch. Hier setzt die vorliegende Studie an.

#### Zielsetzungen

Diese Studie versucht mittels eines Mixed-Methods Design (Schwerpunkt in diesem Beitrag: 3 qualitative Fragen) die Erfahrungen mit dem Medium der Video-Psychotherapie (und auch Sprachtelefonie) im ersten Jahr der Covid-19 Pandemie in einer Querschnittstudie mit zwei Erhebungszeitpunkten zu erfassen. Folgende zentrale Fragen sollen beantwortet werden:

1. Auf welche therapeutischen Möglichkeiten muss bei Sprach- und Video-Psychotherapie verzichtet werden?
2. Welche neuen Möglichkeiten bietet das Setting Video-Psychotherapie?
3. Wann sollte ein therapeutisches Angebot über Distanz empfohlen werden?

## Studiendesign, Erhebungszeitpunkte und Stichprobe

Psychotherapeut_innen der Berufsliste des Bundesministeriums für Soziales, Gesundheit, Pflege und Konsumentenschutz (BMSGPK) wurden zu zwei Erhebungszeitpunkten (s_1_: 23.04.–31.05.2020, s_2_: 26.11.2020–03.01.2021^1^) über eine E‑Mail zu einer Online-Befragung über SoSci Survey eingeladen. Der Fragebogen^2^ kombinierte offene und geschlossene Fragen. Die Einladung zum zweiten Erhebungszeitpunkt wurde nur an jene Psychotherapeut*innen geschickt, welche beim ersten angaben, dass sie erneut befragt werden wollten. Die gleichen Therapeuten*innen wurden somit an zwei Erhebungszeitpunkten eingeladen, nicht alle nahmen jedoch zu beiden Zeitpunkten teil. Die Daten beider Erhebungszeitpunkte wurden daher nicht den jeweils gleichen Personen zugeordnet. Es handelt sich somit um einen Querschnitt zu zwei Erhebungszeitpunkten.

Für die Erhebung wurden im Fragebogen folgende Begriffe unterschieden:

1. Persönlicher Kontakt in der Praxis: Mit dem persönlichen Kontakt in der Praxis sind jene Klient_innen‑/Patient_innenkontakte gemeint, die vor Ort in der eigenen Praxis im unmittelbaren, direkten und persönlichen Kontakt geschehen. Hierunter fallen auch alle Hausbesuche, wo ein direkter, physischer Klient_innen‑/Patient_innenkontakt gegeben ist und nicht mittels Sprach- oder Videotelefonie übertragen wird.
2. Sprachtelefonie: Unter Sprachtelefonie sind jene Klient_innen‑/Patient_innenkontakte gemeint, die über ein Telefongespräch oder Telefonat geschehen. Zentral ist hier die reine Sprachkommunikation mit Audiomaterial über ein technisches Medium zwischen Psychotherapeut_in und Klient_in/Patient_in, ohne dass sich beide über ein Bild sehen oder im realen, physischen, persönlichen und unmittelbaren Kontakt sind.
3. Videotelefonie: Unter Videotelefonie sind jene Klient_innen‑/Patient_innenkontakte gemeint, die über ein Telefongespräch oder Telefonat geschehen, wo zusätzlich bewegtes Bildmaterial übermittelt wird. Zentral ist hier die Sprachkommunikation mit Audio und Videomaterial über ein technisches Medium zwischen Psychotherapeut_in und Klient_in/Patient_in, ohne dass beide im realen, physischen, persönlichen und unmittelbaren Kontakt sind.

Es wurde ein breites Spektrum an soziodemografischen und anderen Variablen erhoben, z. B. Alter, Geschlecht, Familienstand, Praxisstandort, im Haushalt lebende Kinder, Intensität der Kinderbetreuung und des Home Schoolings, psychotherapeutisches Fachspezifikum, Jahr der Zulassung als Psychotherapeut, Art und Umfang der Beschäftigung aktuell, aktuelle Nutzung von Sprach- oder Videotelefonaten, Erfahrungen und Zufriedenheit im Kontakt über Sprach- sowie Videotelefonie, aber auch im persönlichen, direkten Kontakt in der Praxis. Es wurde auch erhoben, wie häufig sich die teilnehmenden Psychotherapeut_innen bezogen auf den Zeitraum einer Woche am Ende eines Arbeitstags ermattet, müde und erschöpft fühlten und wie gut und klar sie aktuell ihre beruflichen Tätigkeiten und ihre familiären Aufgaben von Ihrer Freizeit abgrenzen konnten. Zusätzlich kam es zur Angabe in welchem Ausmaß sich die Themen in den psychotherapeutischen Prozessen bei den Klient_innen/Patient_innen verändert hätten und Sprach- und Videotelefonate wurden Polaritäten zugeordnet. Diese Ergebnisse wurden bereits in anderen Studien dargestellt (Höfner et al. 2021b).

Mittels offener Fragen wurden die teilnehmenden Psychotherapeut_innen hinsichtlich der neuen therapeutischen Möglichkeiten bzw. Einschränkungen durch den Einsatz von Sprach- oder Videotelefonie befragt. Es wurde auch erhoben, ob sie Sprach- oder Videotelefonie für die therapeutische Arbeit nach der Krise empfehlen würden und welches Medium sie eher bevorzugen. Weitere Themen waren die Erfahrungen mit diesen Online-Tools und der Erreichbarkeit Patient_innen‑/Klient_innengruppen mit spezifischen Diagnosen^3^.

Für den vorliegenden Beitrag wurden diese offenen Fragen inhaltsanalytisch ausgewertet. Die zentralen Zielsetzungen bestehen in einer ersten systematischen Bestandsaufnahme der Erfahrungen mit diesen Medien in der aktuellen Situation und in der Entwicklung von Hypothesen und Anregungen für weitere Untersuchungen.

In Österreich sind durch das BMSGPK aktuell 23 psychotherapeutische Verfahren anerkannt, wobei diese vier Grundströmungen (psychodynamisch, humanistisch, systemisch und verhaltensorientiert) zugeordnet werden (BMSGPK 2020). Die humanistische Orientierung ist in der vorliegenden Untersuchung, verglichen mit der Psychotherapeut_innenliste des BMSGPK mit rund 47 % zu s_1_ bzw. rund 51 % zu s_2_ überrepräsentiert, die anderen Strömungen sind ausreichend vertreten. Beim ersten Erhebungszeitpunkt s_1_ liegen 743 (79,6 % Frauen) und beim zweiten Erhebungszeitpunkt s_2_ liegen 212 (74,5 % Frauen) vollständig ausgefüllte Online-Fragebögen vor. Median und Modus der erhobenen Altersangaben liegen bei s_1_ und bei s_2_ in der Kategorie 50–54 Jahre. Die Teilnahme an der Befragung war freiwillig. Sagerschnig und Tanios (2017) zeigten, dass 71,6 % der österreichischen Psychotherapeut_innen Frauen sind. Das Durchschnittsalter beträgt 55,7 Jahre, wobei das Durchschnittsalter der Frauen 54,8 Jahre beträgt und jenes der Männer 58 Jahre. Es zeigte sich laut Autor_innen im Betrachtungszeitraum 2012 bis 2015 ein kontinuierlicher Anstieg im Durchschnittsalter. Abgesehen von der Clusterverteilung entspricht die Zusammensetzung in zentralen Merkmalen (Alter, Geschlecht) somit weitgehend den Verteilungskennwerten österreichischer Psychotherapeut_innen.

Tab. 1 zeigt ausgewählte soziodemographische und weitere Variablen zur Stichprobenbeschreibung.

**Table 1:** 

		s_1_ (23.04.–31.05.2020)		s_2_ (26.11.2020–03.01.2021)	
		*N*	%	*N*	%
*Geschlecht*	Weiblich	582	78,3	158	74,5
	Männlich	149	20,1	54	25,5
	Divers	1	0,1	0	0
	Fehlender Eintrag	11	1,5	0	0
*Alter*	25–29 Jahre	1	0,1	0	0
	30–34 Jahre	18	2,4	4	1,9
	35–39 Jahre	51	6,9	8	3,8
	40–44 Jahre	66	8,9	13	6,1
	45–49 Jahre	121	16,3	33	15,6
	50–54 Jahre	161	21,7	49	23,1
	55–59 Jahre	123	16,6	42	19,8
	60–64 Jahre	109	14,7	40	18,9
	65–69 Jahre	47	6,3	11	5,2
	> 69 Jahre	35	4,7	12	5,7
	Fehlender Eintrag	11	1,5	0	0
Psychotherapeutische Orientierung	Psychodynamisch	149	20,1	46	21,7
	Humanistisch	352	47,4	109	51,4
	Systemisch	149	20,1	37	17,5
	Verhaltenstherapeutisch	63	8,5	18	8,5
	Fehlender Eintrag	30	4,0	2	0,9

*s*_*1*_ Erhebungszeitpunkt 1, *s*_*2*_ Erhebungszeitpunkt 2, *N* Stichprobengröße, *%* Prozentuelle Angabe bezogen auf die Gesamtstichprobe

## Auswertung

Die Auswertungen der offenen Fragen erfolgten qualitativ inhaltsanalytisch nach Mayring (2015). Im Rahmen der strukturierten Inhaltsanalyse wurde eine induktive Kategorisierung vorgenommen, die sich an den im Abschnitt „Zielsetzungen“ der vorliegenden Arbeit skizzierten Fragestellungen orientiert. Die Hauptergebnisse nach der Kategorienbildung sind in den folgenden Tabellen dargestellt^4^.

### Ergebnisse

#### Neue Möglichkeiten durch den Einsatz von Sprach- oder Videotelefonie

Auf die Frage „Eröffnen sich für Sie neue therapeutische Möglichkeiten mit Sprach- oder Videotelefonie? Wenn ja, welche?“ antworten beim ersten Erhebungszeitpunkt (s_1_ mit *n* = 743 Befragten) 206 Personen (28 %) mit „nein“, beim zweiten Erhebungszeitpunkt (s_2_ mit *n* = 212 Befragten) sind es nur noch 40 Personen (19 %). Beim ersten Erhebungszeitpunkt sehen 403 Personen (54 %) neue Möglichkeiten therapeutischen Arbeitens gegeben, bei s_2_ sind es 156 Personen (74 %). Zudem berichten 58 (s_1_) bzw. 31 Personen (s_2_), dass sie Sprach- oder Videotelefonie als zuweilen hilfreiche oder notwendige Alternative der Kontaktaufnahme erkennen bzw. bessere Erfahrungen damit gemacht haben als ursprünglich erwartet, diese Antworten beinhalten aber keinen Hinweis darauf, dass darüber hinaus neue Möglichkeiten entstanden seien. (Die Auswertungskategorien sind in Tab. 2 dargestellt).

**Table 2:** 

Kategorie	Unterkategorie	s_1_	s_2_	Ankerbeispiel
*Therapeutischer Prozess*	–	**246**	**77**	**–**
	Kontinuität	108	32	„Betreuung kontinuierlicher möglich, wenn Pat öfter verreist bzw. zwei Wohnsitze“ (156 ^a^, EA ^b^)
	Persönliche Öffnung	42	23	„Körperliche Distanz und Wegfall von Blickkontakt, bei telefonischem Setting (=Sprachtelefonie) erleichtert manchen Patient**innen Zugang zu Themen, die zum Beispiel durch Schamgefühle abgewehrt werden.“ (892, EA)
	Einblick in privates Umfeld	40	9	„Nachdem der Klient zuhause im eigenen Umfeld ist während des Telefonats, können hier auch speziell konkrete Alltagsthemen besser erfasst werden/gleichzeitig neue Verhaltensmöglichkeiten direkter transferiert werden.“ (578, IG)
	Fokus	29	5	„Telefon sehr gute Erfahrungen, meist extrem fokussiertes Arbeiten“ (427, IG)
	Sonstige	27	9	–
*Therapeutischer Rahmen*	–	**162**	**75**	**–**
	Örtliche/zeitliche Flexibilität	137	62	„Zeitliche Flexibilität höher; Einsatz möglich im In- und Ausland nicht abhängig von persönlichem Erscheinen in Praxis; in Krisenzeiten schnell und funktionabel einsetzbar“ (399, IG)
	Vertraute Umgebung	19	6	„Für einige Patient*innen war es angenehm im eigenen Zuhause zu sein (der eigene Wohlfühlraum)“ (96, IP)
	Vereinfachte Bedingungen	6	7	„Praxisraum Miete, Vorbereitung des Raumes etc.“ (556, KIP)
*Kontakt*	–	**24**	**7**	–
	Nähe/Intimität	10	2	„Ja: Es kann u. U. eine besondere Form der Intimität/Nähe entstehen, die für den therapeutischen Prozess förderlich ist“ (240, PA)
	Erhalten/Stärken d. Beziehung	7	0	„Therapeutische Beziehung kann gehalten werden.“ (251, SF)
	Intensivierung Wahrnehmung	5	5	„Ich nehme Resonanzphänomene an mir selber intensiver wahr“ (1013, PD)
	Sonstige	2	0	–

Anmerkungen:

*EA* Existenzanalyse, *IG* Integrative Gestalttherapie, *IP* Individualpsychologie, *KIP* Katathym Imaginative Psychotherapie, *PA* Psychoanalyse, *PD* Psychodrama, *SF* Systemische Familientherapie

^a^ Fragebogen-Code zur Identifizierung des Zitats

^b^ Psychotherapeutisches Verfahren der Therapeut*in

Jene 403 Personen aus der Stichprobe von 720 Befragten, die auf die Frage nach neuen Möglichkeiten mit Sprach- und Videotelefonie (s_1_) beschreiben, dass sie solche erkennen und jene 156 Personen aus der Stichprobe (s_2_), sind wie in Tab. 2 ersichtlich in den Antwortkategorien „Therapeutischer Prozess“, „Therapeutischer Rahmen“ und „Kontakt“ eingeschlossen. Manche Befragten geben aufgrund der offenen Fragestellung Antworten in mehreren Kategorien (siehe Tab. 2).

Neue Möglichkeiten werden vor allem in Bezug auf den **therapeutischen Prozess** (s_1_: 246, s_2_: 77 Nennungen) beschrieben. Allen voran steht dabei die erhöhte Kontinuität (s_1_: 108, s_2_: 32 Nennungen), da Termine auch stattfinden können, wenn eine Person sich gerade nicht in die Praxis begeben kann. Aufgefallen sind einigen Therapeut*innen auch eine vermehrte persönliche Öffnung (s_1_: 42, s_2_: 23 Nennungen) sowie erkenntnisreiche Einblicke ins private Umfeld (s_1_: 40, s_2_: 9 Nennungen) mancher Patient*innen. Manche erleben das Arbeiten in dieser Weise als besonders fokussiert (s_1_: 29, s_2_: 5 Nennungen).

Der **therapeutische Rahmen** (s_1_: 162, s_2_: 75 Nennungen) beinhaltet vor allem sehr viele Nennungen im Bereich der örtlichen und zeitlichen Flexibilität (s_1_: 162, s_2_: 75 Nennungen). Für manche ist die vertraute Umgebung (s_1_: 19, s_2_: 6 Nennungen), in der sich die Patient_innen befinden, eine Bereicherung. Einzelne nennen auch vereinfachte Bedingungen, also Aspekte der Effizienz (s_1_: 6, s_2_: 7 Nennungen).

Nur selten werden neue Möglichkeiten im Bereich des **Kontakts** genannt (s_1_: 24, s_2_: 7 Nennungen). Bisweilen ist eine besondere Nähe oder Intimität spürbar (s_1_: 10, s_2_: 2 Nennungen). Auch zum Erhalten oder Stärken der Beziehung (s_1_: 7, s_2_: 0 Nennungen) bzw. zu einer Intensivierung der Wahrnehmung (s_1_: 5, s_2_: 5 Nennungen) äußern sich einzelne Befragte.

#### Einschränkungen bei therapeutischen Möglichkeiten aufgrund von Sprach- oder Videotelefonie

Eine weitere offene Frage bezieht sich auf die erlebten Einschränkungen im Zusammenhang mit Sprach- und Videotelefonie: „Auf welche Methoden/Vorgangsweisen (therapeutische Möglichkeiten) mussten Sie aufgrund des Einsatzes von Sprach- oder Videotelefonie verzichten?“. Dabei geben beim ersten Erhebungszeitpunkt nur vereinzelt Personen an, dass sie auf alle Methoden/Vorgangsweisen verzichten müssen. 6,7 % der Befragten müssen hingegen nach ihren Angaben auf keine Methoden/Vorgangsweisen verzichten. Zum zweiten Erhebungszeitpunkt gibt niemand mehr an, auf alle Methoden/Vorgangsweisen verzichten zu müssen, 12 der befragten Therapeut_innen meinen, auf nichts verzichten zu müssen.

Hier beziehen sich bei der Beantwortung der Frage sehr viele der Befragten auf konkrete therapeutische Techniken, andere beschreiben Schwierigkeiten bzw. Einschränkungen in den Bereichen, die im Zusammenhang mit den neuen Möglichkeiten (vgl. die oben dargestellten Ergebnisse) ebenfalls thematisiert wurden (Kontakt, Therapeutischer Prozess, Therapeutischer Rahmen).

Die Ergebnisse zu den konkreten therapeutischen Techniken sind in Tab. 3 dargestellt Ebenso wie die Einschränkungen bzw. Schwierigkeiten in Bezug auf den Kontakt, den therapeutischen Prozess und den therapeutischen Rahmen.

**Table 3:** 

Kategorie	Unterkategorie	s_1_ *n* = 720	s_2_ *n* = 213	Ankerbeispiele
*Szenisches Erleben*	**–**	**375**	**110**	**–**
	Kreative Medien	83	26	„Teilweise auf kreative Medien z. B. Tonarbeit (kommt stark darauf an, was Klient*in zu Hause hat und über Videotelefonie auch bereit ist zeigen zu wollen. Ist oft schon in der Praxis große Hürde, zu Hause oft noch mehr.“ (399 ^a^, IG ^b^)
	Aufstellungen	81	22	„Aufstellungsarbeit“ (731, IG)
	Stuhlarbeit	56	15	„Arbeit mit leerem Stuhl“ (367, IG)
	Aufstellungsbrett	44	14	„Aufstellungsarbeit am Brett“ (280, HY)
	Imaginationen	38	10	„Imaginationen sind schwerer zu begleiten, da bei mir das Bild nicht so ‚auftaucht‘“ (584, EA)
	Rollenspiele	33	5	„Rollenspiele“ (1597, KIP)
	Arbeit im Raum	17	6	„Einbeziehung des Raumes“ (579, IT)
	Sonstige	23	12	–
*Selbsterleben*	–	**245**	**54**	**–**
	Traumaspezif. Methoden	87	28	„Aufarbeitung von psychischen Traumata, da die Klienten vor allem bei der Sprachtelefonie zu wenig geschützt und geleitet und aufgefangen werden können.“ (851, EA)
	Trance/Hypnose	33	7	„Tranceinduktion“ (1055, HY)
	Symbole	29	5	„Nutzen von Gegenständen als Symbole“ (293, KBT)
	Spielen	28	5	„Spiele und Aktivitäten mit Kindern“ (915, SF)
	Sonstige	68	9	–
*Körpererleben*	–	**172**	**62**	**–**
	Körperinterventionen	127	46	„Körperorientierter Teil der Therapie ist nur sehr eingeschränkt verfügbar“ (914, oE)
	Entspannung	17	6	„Auch Entspannungstechniken finde ich über Telefon schwierig“ (1246, IG)
	Bewegung	12	8	„Bewegungszentriertes Vorgehen“ (1483, IT)
	Körperwahrnehmung	13	1	„Körperwahrnehmung seitens der Pat. war nur eingeschränkt möglich“ (489, KBT)
	Biofeedback	3	0	„Biofeedback“ (901, SF)
*Sprachliches Erleben*	–	**87**	**21**	**–**
	Flipchart/Modelle Skizzieren	27	4	„Flipchart fehlt hier- also Möglichkeit, etwas aufzuzeichnen oder aufzuschreiben habe aber auch viele Dokumente, die ich als ‚hardcopy‘ an Kl weiterschicken kann“ (100, SF)
	Eingehen auf Körperausdruck	12	3	„Bezugnahme auf diverse körperliche Reaktionen/Handlungen nicht oder nur sehr eingeschränkt möglich.“ (194, SF)
	Konfrontation	12	5	„Größte Vorsicht mit Konfrontationen“ (1160, IG)
	Interventionen mit diversen Karten	10	2	„Arbeit mit Moderationskarten um etwa Cluster, Priorisierungen von Themen visuell darzustellen“ (161, EA)
	Sonstige	26	7	–

Anmerkungen:

*oE* ohne Eintragung, *EA* Existenzanalyse, *IG* Integrative Gestalttherapie, *HY* Hypnosepsychotherapie, *IT* Integrative Therapie, *KBT* Konzentrative Bewegungstherapie, *KIP* Katathym Imaginative Psychotherapie, *SF* Systemische Familientherapie

^a^ Fragebogen-Code zur Identifizierung des Zitats

^b^ Psychotherapeutisches Verfahren der Therapeut*in

Die häufigsten Nennungen zu Einschränkungen bei *therapeutischen Techniken* werden zu beiden Erhebungszeitpunkten im Bereich „**Szenisches Erleben**“ berichtet (s_1_: 375, s_2_: 110 Nennungen). Vor allem kreative Medien (s_1_: 83, s_2_: 26 Nennungen), Aufstellungen mit und ohne Brett (s_1_: 125, s_2_: 36 Nennungen), Stuhlarbeit (s_1_: 56, s_2_: 15 Nennungen), Imaginationen (s_1_: 38, s_2_: 10 Nennungen), Rollenspiele (s_1_: 33, s_2_: 5 Nennungen) und die Arbeit im Raum (s_1_: 17, s_2_: 6 Nennungen) erleben Therapeut*innen zu beiden Erhebungszeitpunkten entweder eingeschränkt oder nicht durchführbar, wobei hier der Lerneffekt im Laufe der Zeit deutlich ins Auge springt. Die detaillierten Zahlen finden sich in Tab. 3.

Interventionen im Bereich „**Selbsterleben**“ werden ebenso häufig als eingeschränkt erlebt (s_1_: 245, s_2_: 54 Nennungen). Am häufigsten ist dies bei traumaspezifischen Methoden der Fall, wobei mehr als die Hälfte der Antworten sich konkret auf bilaterale Stimulation beziehen (s_1_: 87, s_2_: 28 Nennungen). Auch Trance/Hypnose (s_1_: 33, s_2_: 7 Nennungen), die Arbeit mit Symbolen (s_1_: 29, s_2_: 5 Nennungen) bzw. das Spielen (s_1_: 28, s_2_: 5 Nennungen) werden von einigen Therapeut_innen als eingeschränkt oder unmöglich beschrieben. (vgl. Tab. 3).

Im Bereich „**Körpererleben**“ (s_1_: 172, s_2_: 62 Nennungen) beschreiben die Befragten den Verzicht auf Köperinterventionen mit oder ohne Berührung (s_1_: 127, s_2_: 46 Nennungen). Auch Entspannungstechniken (s_1_: 17, s_2_: 6 Nennungen), Interventionen mit Bewegung (s_1_: 12, s_2_: 8 Nennungen) oder zur Körperwahrnehmung der Klient_innen (s_1_: 13, s_2_: 1 Nennung) und Biofeedback (s_1_: 3, s_2_: 0 Nennungen) finden Erwähnung. (vgl. Tab. 3).

Interventionen im Bereich „**Sprachliches Erleben**“ werden am wenigsten eingeschränkt erlebt (s_1_: 87, s_2_: 21 Nennungen). Hier geht es vor allem um Interventionen, die Material benötigen wie das Darstellen auf Papier oder Kärtchen. Allerdings wird dies im zweiten Erhebungszeitpunkt deutlich seltener thematisiert (s_1_: 37, s_2_: 6 Nennungen). Ein weiterer Bereich betrifft Rückmeldungen oder Konfrontation (s_1_: 24, s_2_: 8 Nennungen), welche schwieriger sind. (vgl. Tab. 3).

Abseits von therapeutischen Methoden wird die therapeutische Vorgehensweise in Bezug auf den Kontakt, den therapeutischen Prozess und den therapeutischen Rahmen als eingeschränkt erlebt (vgl. Tab. 4). Am häufigsten thematisieren die Befragten, dass sie beim Einsatz von Sprach- und Videotelefonie auf Aspekte des **Kontakts** verzichten müssen (s_1_: 214, s_2_: 54 Nennungen). Dies betrifft in erster Linie die visuelle Wahrnehmung von Körperausdruck, Mimik und Gestik der Patient_in (s_1_: 99, s_2_: 24), aber auch die Wahrnehmung von Emotionen (s_1_: 21, s_2_: 4) sowie von Übertragung/Gegenübertragung (s_1_: 11, s_2_: 4). Die fehlende persönliche Begegnung wird vor allem beim ersten Erhebungszeitpunkt als Einschränkung im Kontakt beschrieben (s_1_: 31, s_2:_ 4). Die eingeschränkte nonverbale Interaktion ist zum zweiten Erhebungszeitpunkt stärker gewichtet als zum ersten (s_1_: 15, s_2_: 20). Auffallend ist zudem, dass der fehlende Blickkontakt nur in der ersten Befragung Erwähnung findet (s_1_: 13, s_2_: 0).

**Table 4:** 

Kategorie	Unterkategorie	s_1_ *n* = 720	s_2_ *n* = 213	Ankerbeispiele
*Kontakt*	–	**214**	**54**	**–**
	Visuelles Wahrnehmen	99	24	„Mimik manchmal durch schlechte Bildqualität schwer einzuschätzen.“ (177 ^a^, SF ^b^)
	Persönliche Begegnung	31	4	„Ich musste auf eine wesentliche Erlebens‑, Verhaltens- und Beziehungsebene verzichten: die der ganzheitlich empfundenen körperlichen Präsenz. Die persönliche Begegnung ist schwer in Worte zu fassen und durch nichts zu ersetzen.“ (1313, IP)
	Emotionales Wahrnehmen	21	4	„Mir fällt es oft schwerer Affekte zu identifizieren. Wenn ich die Menschen per Telefon nicht sehe, ist das besonders schwer, aber auch die oft schlechte Bildqualität der Videotelefonie erschweren das.“ (199, DG)
	Nonverbale Interaktion	20	15	„Bei starker Emotionalität weniger Möglichkeit Nähe und Sicherheit körperlich zu signalisieren.“ (1521, VT)
	Blickkontakt	13	0	„zB direkte Augenkontakte sind rein technisch nicht wirklich möglich – entweder ich blicke in die Kamera oder auf den Screen“ (1035, PT)
	Wahrnehmen von Übertragung/Gegenübertragung	11	4	„Als tiefenpsychologische Therapeutin fehlt zu einem großen Teil Übertragung und Gegenübertragung.“ (334, IP)
	Sonstige Einschränkungen in der Gestaltung des Kontakts	19	3	„Das ‚Ankommen lassen‘: Ruhige Musik, Getränke anbieten usw.“ (161, EA) „z. B. das Reichen eines Taschentuches.“ (687, EA)
*Therap. Prozess*	**–**	**42**	**9**	**–**
	Oberflächlichkeit	19	3	„Es ist ungleich schwerer emotionale Themen über Videotelefonie anzuregen bzw. zu erfassen – ist möglich dauert aber im Videogespräch länger muss vielmehr verbal unterstützt werden.“ (1164, DG)
	Schweigen	10	5	„Ein Schweigen in der Therapiesituation ist völlig normal, am Telefon wirkt das völlig anders, teils irritierend.“ (1154, PT)
	Sonstige	13	2	–
*Therap. Rahmen*	–	**33**	**7**	**–**
	Atmosphäre	15	2	„‚Atmosphärisch‘ ist es ein gewaltiger Unterschied“ (408, PT)
	Störfaktoren	14	4	„Großer Störfaktor ist die Tatsache, dass der Patient selbst für einen sicheren, ungestörten Ort sorgen muss (Bsp.: jederzeit kann der Partner/die Partnerin oder ein Kind die Sitzung stören).“ (524, TP)
	Datensicherheit	2	0	„Datenschutzrechtliche Bedenken“ (549, PD)
	Weg	2	1	„Den Patienten geht der Weg in die Praxis und von der Praxis nach Hause ab (Vor- und Nachbearbeitung des Erlebten in der Therapiesitzung)“ (524, TP)

Anmerkungen:

*DG* Dynamische Gruppenpsychotherapie, *EA* Existenzanalyse, *IP* Individualpsychologie, *PD* Psychodrama, *PT* Personenzentierte Therapie, *SF* Systemische Familientherapie, *TP* Transaktionsanalytische Psychotherapie, *VT* Verhaltenstherapie

^a^ Fragebogen-Code zur Identifizierung des Zitats

^b^ Psychotherapeutisches Verfahren der Therapeut*in

Im **therapeutischen Prozess** (s_1_: 42, s_2_: 9 Nennungen) erleben zum ersten Erhebungszeitpunkt deutlich mehr Therapeut_innen eine gewisse Oberflächlichkeit (s_1_: 19, s_2_: 3 Nennungen). Die Schwierigkeit, Schweigen bei dieser Art des Kontakts für längere Zeit auszuhalten, thematisieren 10 (s_1_) bzw. 5 Personen (s_2_).

Der **therapeutische Rahmen** ist für manche Befragte atmosphärisch verändert (s_1_: 15, s_2_: 2 Nennungen). Es gibt Störfaktoren (s_1_: 14, s_2_: 4 Nennungen) wie die fehlende Privatsphäre einiger Patient_innen zuhause oder technische Schwierigkeiten. Datenschutzrechtliche Bedenken werden kaum geäußert (s_1_: 2, s_2_: 0 Nennungen). Der fehlende Weg zur Psychotherapie findet vereinzelt Erwähnung (s_1_: 2, s_2_: 1 Nennung/en).

### Weitere Empfehlung von Sprach- oder Videotelefonie

Zu beiden Erhebungszeitpunkten wurde die Frage gestellt: „Würden Sie Sprach- oder Videotelefonie für die therapeutische Arbeit nach der Krise empfehlen?“ und als Zusatz „Wenn ja, wem?“

In der ersten Befragung meinen 29 Therapeut_innen, dass sie Sprach- oder Videotelefonie uneingeschränkt empfehlen würden, bei der zweiten sind es 17. Ein klares „Nein“ zu einer Empfehlung sprechen 113 (s_1_) bzw. 22 Personen (s_2_) aus. Weiters empfinden viele Befragte den Einsatz von Sprach- oder Videotelefonie als Überbrückung bzw. in Kombination mit persönlichem Kontakt einsetzbar (s_1_: 93, s_2_: 24 Nennungen), einige empfinden es auch lediglich als Ausnahme oder Notlösung (s_1_: 76, s_2_: 28 Nennungen). Viele Therapeut_innen nutzen diese Möglichkeiten zur Krisenintervention (s_1_: 86, s_2_: 13 Nennungen), manche geben an, dass sie diese Form des Kontakts nicht als Psychotherapie beschreiben würden, sondern eher als Beratung, Coaching oder Begleitung (s_1_: 27, s_2_: 6 Nennungen).

Manche Angaben zur Zielgruppe (Zusatzfrage zur Empfehlung „Wenn ja, wem?“) sind sehr allgemein gehalten, etwa wenn Psychotherapie persönlich nicht möglich ist (s_1_: 73, s_2_: 19 Nennungen) (vgl. Tab. 5). Einige geben an, es sei individuell sehr unterschiedlich (s_1_: 14, s_2_: 2 Nennungen). Weiters würden einige Befragte nur mit bereits bekannten Patient_innen ein solches Setting empfehlen (s_1_: 38, s_2_: 4 Nennungen).

**Table 5:** 

Kategorie	Unterkategorie	s_1_	s_2_	Beispiel(e)
*Wenn Psychotherapie persönlich nicht möglich ist*	***–***	**73**	**19**	„Möglich ist dies, wenn ein Kommen nicht möglich ist, aus welchem Grund auch immer.“ (96 ^a^, IP ^b^)
*Individuell bestimmen*	–	**14**	**2**	„Wem, ist individuell zu bestimmen“ (361, PT)
Bereits bekannte Klient*innen	–	**38**	**4**	„Wenn bereits persönliche, gute therapeutische Beziehung sich etabliert hat“ (151, PT)
*Auf Wunsch*	–	**17**	**2**	„Ja. Bei Bedarf jedem, der sich mit der Methode wohl fühlt und wenn der Patient es wünscht.“ (461, VT)
*Immobilität*	–	**46**	**20**	„Ja durchaus für Patienten, die nicht mobil sind.“ (193, VT)
*Entfernung*	–	**183**	**65**	„bei längeren Auslandsaufenthalten“ (151, w, PT) „Neuen Klienten aus entfernten Gebieten, wo die psychotherapeutische Versorgung schlecht ist“ (202, EA)
*Organisatorische Gründe*	–	**64**	**21**	„Bei voller Berufsauslastung oder sonstigen Umständen, in denen eine Face-to-Face-Einheit erheblichen zeitlichen Aufwand“ (254, PT)
*Körperliche Gesundheit*	–	**99**	**33**	„Krankenstand des Klienten ist kein Grund mehr für Absage“ (784, SF)
*Psychische Gesundheit*	–	**119**	**27**	–
	Bei geringer Symptomatik	9	1	„Bedingt; bei eher leichteren Krankheitsbildern bzw. Symptomen“ (162, SF)
	Personen, die niederschwelligen Einstieg brauchen	29	14	„Außerdem für alle, die zuerst unüberwindliche Scheu haben, eine Praxis aufzusuchen.“ (189, oE)
	Angst/Zwang	55	9	„Vielleicht einer Klientin, die auf Grund ihrer Zwangs-Erkrankung kaum schafft, Termine wahrzunehmen, weil sie nicht schafft, rechtzeitig außer Haus zu gehen.“ (116, EA)
	Depression	15	2	„Depressive, die kaum auf die Straße gehen wollen, könnten leichter abgeholt werden“ (639, IG)
	Sonstige	11	1	–

Anmerkungen:

*oE* ohne Eintragung, *EA* Existenzanalyse, *IG* Integrative Gestalttherapie, *IP* Individualpsychologie, *PT* Personenzentierte Therapie, *SF* Systemische Familientherapie, *VT* Verhaltenstherapie

^a^ Fragebogen-Code zur Identifizierung des Zitats

^b^ Psychotherapeutisches Verfahren der Therapeut*in

Empfehlen würden die befragten Therapeut_innen diese Möglichkeit vor allem bei großer Entfernung (s_1_: 183, s_2_: 65 Nennungen), aber auch bei körperlichen oder gesundheitlichen Einschränkungen (s_1_: 99, s_2_: 33 Nennungen), aus organisatorischen Gründen (s_1_: 64, s_2_: 21 Nennungen) sowie bei Immobilität (s_1_: 46, s_2_: 20 Nennungen). Die zweithäufigste Kategorie ist zudem die psychische Gesundheit (s_1_: 119, s_2_: 27 Nennungen). So wird Sprach- oder Videotelefonie für Menschen mit depressiven Störungen (s_1_: 15, s_2_: 2 Nennungen) und Angsterkrankungen (s_1_: 55, s_2_: 9 Nennungen) empfohlen und auch allgemein für Personen, die einen niederschwelligen Einstieg brauchen (s_1_: 29, s_2_: 14 Nennungen). Vereinzelt wird auch beschrieben, dass vor allem bei geringer Symptomatik (s_1_: 9, s_2_: 1 Nennungen) auch ein Setting per Sprach- oder Videotelefonie denkbar wäre. (vgl Tab. 5).

### Bevorzugung von Sprach- oder Videotelefonie

In der zweiten Erhebung wurde erfragt, weshalb entweder Sprach- oder Videotelefonie bevorzugt eingesetzt werde: „Wenn ein face-to-face-Kontakt nicht möglich ist: Bevorzugen Sie Sprach- oder Videotelefonie?“.

Die Vorteile von Sprachtelefonie sehen die Therapeut_innen vor allem darin, dass es sich um eine technisch einfache und zuverlässige Form der Kommunikation handelt (24 Nennungen), zudem werde Sprachtelefonie häufig von den Patient_innen bevorzugt (11 Nennungen). Einige Psychotherapeut_innen stellen fest, dass sie sich bei diesem Setting besser konzentrieren können als bei der Anwendung von Videotelefonie (10 Nennungen) und dass sie über das ausschließliche Hören sehr viel wahrnehmen können (9 Nennungen). Die Sprachtelefonie ist außerdem für alle Beteiligten ein gut bekanntes Medium (9 Nennungen), die Kommunikation sei auch weniger anstrengend bzw. entspannter (7 Nennungen). Manche betonen explizit, dass es sie störe, sich selbst durchgehend zu sehen, wie das bei der Videotelefonie der Fall ist (5 Nennungen). Der Wegfall des visuellen Aspekts bringe darüber hinaus mehr räumliche Unabhängigkeit (4 Nennungen) und schütze die Privatsphäre (3 Nennungen). Für manche fühle sich diese Art der Kommunikation auch persönlicher oder natürlicher an (vgl. Tab. 6).

**Table 6:** 

Kategorie	Unterkategorie	Anzahl
*Sprachtelefonie, weil …*	Technisch einfacher/zuverlässiger	24
	Von Klient*innen bevorzugt	11
	Besser konzentrieren	10
	Gut bekannt	9
	Über Hören viel wahrnehmen	9
	Weniger anstrengend/entspannter	7
	Selbst sehen stört	5
	Persönlicher	5
	Räumliche Unabhängigkeit	4
	Von Therapeut*in bevorzugt	3
	Schutz der Privatsphäre	3
	Flexibel einsetzbar	2
	Klient*in mit sich in Kontakt	1
	Besseres inneres Bild	1
	Natürlicher	1
	Datensicherheit	1
	Übersättigung Videotelefonie	1
*Videotelefonie weil, …*	Visuelle Wahrnehmung	89
	Kontakt/Beziehung	19
	Näher am persönlichen Setting	15
	Emotionen	10
	Mehr Möglichkeiten	8
	Weniger anstrengend	7
	Pausen besser aushaltbar	2
	Verbindlicher	1
	Von Klient*innen bevorzugt	1

Für die Videotelefonie spreche vor allem, dass über die visuelle Wahrnehmung deutlich mehr Informationen zur Patient_in vorhanden seien (89 Nennungen). Einige Therapeut_innen empfinden in dem Zusammenhang auch den Kontakt oder die Beziehung als besser (19 Nennungen) und erleben diese Art des Kontakts als näher am persönlichen Setting (15 Nennungen). Emotionen seien deutlicher erkennbar und leichter einzubeziehen (10 Nennungen), ganz allgemein gebe es in diesem Setting mehr Möglichkeiten (8 Nennungen) und es sei auch weniger anstrengend als Sprachtelefonie (7 Nennungen). Pausen erleben einzelne Therapeut_innen als leichter aushaltbar (2 Nennungen). Nur einmal wird genannt, dass Patient_innen Videotelefonie gegenüber Sprachtelefonie bevorzugen (vgl. Tab. 6).

## Diskussion

In dieser Studie wurden die Erfahrungen von österreichischen Psychotherapeut_innen mit Sprach- und Video-Psychotherapie im Verlauf der Covid-19 Pandemie erfasst. Die hier präsentierten qualitativen Analysen erbringen ein differenziertes Bild von Möglichkeiten, aber auch von Einschränkungen in der psychotherapeutischen Arbeit mit diesen Kommunikationsmedien.

Viele der erlebten Einschränkungen werden in Bezug zu den jeweiligen Therapiemethoden erlebt, die neuen Möglichkeiten werden vorwiegend im Bereich der Flexibilisierung von Settingaspekten gesehen. Vieles ist dabei ambivalent; spezifische Vorteile (z. B. therapeutische Nähe in der Intimität des Privatbereichs der Klient_innen) stehen spezifischen Nachteilen (z. B. fehlende Abgrenzung vom Alltagsleben) gegenüber. Nach den Einschätzungen eignet sich Video-Psychotherapie einerseits für bestimmte Einschränkungen in der Wahl des Settings (geografische Distanz), bei den Klient_innen (eingeschränkte Mobilität, Niederschwelligkeit etc.), oder auch für bestimmte Störungsbilder (in mittelgradigen Ausprägungen). Viele der Einschätzungen haben sich über den Erfahrungszeitraum im ersten Jahr der Pandemie verändert bzw. häufig auch verbessert. Hier zeigt sich eindeutig ein positiver und differenzierter Lernprozess mit dieser Form der psychotherapeutischen Arbeit.

Vor der Pandemie wurde Video-Psychotherapie nur von einer kleineren und interessierten Gruppe von Psychotherapeut_innen akzeptiert, was dadurch belegt wird, dass 41 % der Psychotherapeut_innen angeben, vor der Pandemie zumindest eher negativ zur Möglichkeit von dieser Art von Psychotherapien gestanden sind und dass nur 31 % diese Option zumindest eher positiv sahen (Uhl et al. 2020). Im Zuge der Pandemie halbierte sich laut den Autor_innen der Anteil der Negativurteile auf 23 % und der Anteil der Positivurteile verdoppelte sich auf 66 %.

Die in der vorliegenden Studie erfassten Einschätzungen beziehen sich hingegen auf eine große und auch in wichtigen Merkmalen (Geschlecht, Alter) weitgehend repräsentative bzw. zumindest sehr breit gestreute Gruppe von Psychotherapeut_innen; sie zeigen daher die Möglichkeiten und Grenzen des Mediums für die gesamte Psychotherapie recht gut auf. Hier erscheint uns ein qualitativer Zugang unerlässlich, weil es nur so möglich ist, die vielschichtigen und vor allem subtilen Erfahrungen und Unterschiede zur Präsenztherapie zu erfassen. Für die Gruppe von Psychotherapeut_innen, die vor der Pandemie so gearbeitet haben wurde die Effektivität von Video-Psychotherapie bereits mehrfach bestätigt (mit in etwa gleich großen Effektstärken wie für die Präsenztherapie). Ob dies auch für die gesamte Psychotherapie, also auch für Strömungen außerhalb der CBT-Ansätze, gilt, wäre noch zu zeigen.

Schulenspezifische Unterschiede und Erfahrungen bzw. Strömungen werden bei diesem Medium jedoch offensichtlich. Es greift wohl zu kurz, wenn in der bisher größten Metaanalyse die positiven Effekte von Video-Psychotherapie darauf zurückgeführt werden, dass vorwiegend therapeutische Angebote, wo die therapeutische Beziehung weniger Rolle spielt, umgesetzt werden (Fernandez et al. 2021). Ganz im Gegenteil: Mit Bezug auf die Forschung von allgemeinen Wirkfaktoren ist wohl anzunehmen, dass auch in der Video-Psychotherapie die „virtualisierte“ therapeutische Beziehung den zentralen Wirkfaktor darstellt. Hier sind Unterschiede in Menschenbildern und in therapeutischen Strömungen zu berücksichtigen, die letztlich auch weitreichende Konsequenzen für die Video-Psychotherapie haben. Darüber hinaus eignen sich die Konzepte und Techniken der therapeutischen Strömungen in unterschiedlichem Maße für eine Umsetzung über Distanz. Vor dem Hintergrund dieser Überlegungen erfolgte auch eine erste Kategorisierung der qualitativen Fragen, die auf wesentlich unterschiedliche Zugangsweisen im therapeutischen Prozess therapeutischer Schulen abzielt: Die jeweilige Fokussierung auf körperliches Erleben, szenisches Erleben und sprachliches Erleben im Rahmen des aktuellen Selbsterlebens im therapeutischen Prozess erfährt durch mediengestützte Psychotherapie eine wesentliche Veränderung auf methodischer und technischer Ebene.

Unsere Studie liefert hier erste Ergebnisse dazu, die allerdings in weiteren Arbeiten noch vertieft werden sollten. Neuere Befunde zur therapeutischen Beziehung bei Video-Psychotherapie verweisen in diesem Zusammenhang – neben den Prozessmerkmalen der therapeutischen Beziehung – beispielsweise auf die Rolle des Therapieorts (Privatheit, gezielte Trennung des Settings für die therapeutische Arbeit etc.), die Auswirkung der Virtualität auf die Beziehungserfahrung, die erweiterte Möglichkeit der Kontrolle durch die Klient_innen (Abbruch einer Sitzung ist auf Knopfdruck möglich) und auf die möglichen Chancen und Auswirkungen von hybriden Settings hin (Kocsis und Yellowlees 2018).

Relativ klar scheint zu sein, dass sich Video-Psychotherapie besser für Einzelsettings eignet. Gruppenprozesse werden durch das Medium eher eingeschränkt. Aber auch hier gibt es einige gegenteilige Befunde (vgl. de Boer et al. 2021).

### Limitationen der vorliegenden Studie

Auch diese Studie weist einige Limitationen auf, die berücksichtigt werden müssen. Die Datenerhebung im Rahmen einer Online-Erhebung kann zu gewissen Verzerrungen führen, da sich internetaffine Personen möglichweise eher angesprochen fühlen. Dieser Effekt könnte sich über die zwei Erhebungen hinweg sogar noch weiter verstärken. Gleichzeitig ist anzunehmen, dass Psychotherapeut_innen mit persönlichen Erfahrungen mit den neuen Medien sich durch die Studie eher angesprochen fühlten und daher überproportional mitgemacht haben. Wir nehmen daher an, dass die Studie trotz dieser Einschränkungen einen guten Überblick über den Stand der Erfahrungen mit Psychotherapie über Distanz gibt und der hypothesengenerierende Anspruch eingelöst wird. Die Ergebnisse bilden allerdings nur die Perspektive der Psychotherapeut_innen ab; die Erfahrungen der Klient_innen mit diesen Medien war nicht Gegenstand der Studie. Auch die Unterscheidung der Möglichkeiten von Video- und Sprachtherapie könnte in zukünftigen Studien noch weiter ausdifferenziert werden.

### Ausblick

Es stellt sich nun die Frage, wie sich nach dieser intensiven Lernerfahrung während der Pandemie das „neue“ digitale Medium der Video-Psychotherapie weiter verbreitet und etabliert. Lovejoy et al. (2009) beziehen sich dazu auf die „Diffusion of Innovations Theory“ und argumentieren, dass nach einer Phase der „early adopter“ (hier wären das die Therapeut_innen, die bereits vor der Pandemie mit Video-Therapie gearbeitet hatten) die weitere Verbreitung über die schrittweise Überwindung spezifischer Barrieren (ethische Fragen, Infrastruktur, rechtliche Rahmenbedingungen, Akzeptanz durch Therapeut_innen und Klient_innen etc.) erfolgen kann. Dazu kommen noch unterschiedliche Menschenbilder und wissenschaftstheoretische Positionen in der Psychotherapie, die vielleicht die Verbreitung begrenzen (beispielsweise die Technologiekritik in einem radikal humanistischen Menschenbild).

Welche Empfehlungen können nun abschließend für Praktiker_innen gemacht werden? Einfache Empfehlungen greifen derzeit wohl noch zu kurz. Unsere Befunde legen jedenfalls nahe, dass die Arbeit mit Video-Psychotherapie eine intensive Lernerfahrung sein kann, deren Möglichkeiten und Grenzen in den therapeutischen Strömungen zum derzeitigen Zeitpunkt noch nicht voll erkannt bzw. ausgeschöpft sind. Die Auseinandersetzung mit diesem Medium und die Sammlung weiterer persönlicher Erfahrungen sind jedoch angebracht und können zur Weiterentwicklung der Psychotherapie in den unterschiedlichen Strömungen und zur Verbesserung der Patient_innenversorgung beitragen.

## Footnotes

1 Die Bezeichnungen s1/s2 wurden gewählt, um auf den Querschnittcharakter der Studie hinzuweisen.

2 Auf Nachfrage kann der Fragebogen bei den Studienautor*innen angefragt werden. Der Fragebogen zu s_2_ umfasste neben deskriptiven Fragen zur Person auch Fragen zur Veränderung der Arbeitssituation von Psychotherapeut_innen während der COVID-19-Pandemie und zur Veränderung der Themen in den Psychotherapien. Diese Ergebnisse werden an anderer Stelle veröffentlicht (Höfner et al. 2021a, b; Mantl et al. 2021).

3 Es wurde auch erfragt, ob aktuell mit Klient_innen/Patient_innen gearbeitet wird, welche an COVID-19 erkrankt sind bzw. waren und über welche diesbezüglichen Themen in der Psychotherapie gesprochen wird bzw. wie die Reaktionen aus dem psychosozialen Umfeld der Klient_innen/Patient_innen auf die Erkrankung waren. Diese COVID-19-spezifischen Ergebnisse befinden sich noch in der Auswertung.

4 Zitate wurden aus der Beantwortung der Fragebögen wortwörtlich (inklusive möglicher Fehler) übernommen, um ein realitätsgetreues Abbild der Beantwortung zu schaffen.
